# Increased peripheral blood TCD4^+^ counts and serum SP-D levels in patients with chronic paracoccidioidomycosis, during and after antifungal therapy

**DOI:** 10.1590/0074-02760170046

**Published:** 2017-11

**Authors:** James Venturini, Ricardo Souza Cavalcante, Tatiane Fernanda Sylvestre, Rodolfo Ferreira dos Santos, Daniela Vanessa Moris, Lídia Raquel Carvalho, Maria Sueli Parreira de Arruda, Marjorie de Assis Golim, Rinaldo Poncio Mendes

**Affiliations:** 1 Universidade Estadual Paulista, Faculdade de Medicina, Botucatu, SP, Brasil; 2 Universidade Estadual Paulista, Faculdade de Ciências, Bauru, SP, Brasil; 3 Universidade Estadual Paulista, Instituto de Biociências, Botucatu, SP, Brasil

**Keywords:** paracoccidioidomycosis, T-lymphocyte subsets, antifungal agents, pulmonary fibrosis, emphysema, surfactant protein-D

## Abstract

**BACKGROUND:**

The main clinical forms of paracoccidioidomycosis (PCM) are the acute/subacute form (AF) and the chronic form (CF), and they both display considerable clinical variability. The immune responses of PCM patients, during and after treatment, remain neglected, mainly in the case of CF patients, due to the high prevalence of pulmonary sequelae.

**OBJECTIVE:**

To evaluate the distribution of whole blood T cell subsets, serum cytokines, and biomarkers of pulmonary fibrosis in PCM patients, according to the clinical form and at different time points, during the antifungal therapy.

**METHODS:**

Eighty-seven PCM patients, from an endemic area in Brazil, were categorised into groups, according to the clinical form (AF or CF) and the moment of treatment. The peripheral blood T lymphocyte subsets of these patients were analysed using fluorescence-activated cell sorting. The serum levels of cytokines, basic fibroblast growth factor and surfactant protein-D (SP-D) were also analysed.

**FINDINGS:**

In the CF patients, an expansion of the peripheral blood TCD4^+^ cells was observed during the treatment, and this persisted even after two years of antifungal treatment. In addition, these patients showed high serum levels of SP-D.

**CONCLUSION:**

Our findings highlight the immunological changes CF patients undergo, during and after treatment, possibly due to the hypoxia triggered by pulmonary fibrosis and emphysema.

Paracoccidioidomycosis (PCM) is a systemic and granulomatous mycosis caused by thermally dimorphic fungi of the genus *Paracoccidioides* (Teixeira et al. 2009), and is characterised by antigen-dependent immunosuppression (Benard et al. 1996). Incidences of PCM are confined to Latin America, and are especially prevalent in Brazil, Colombia, and Venezuela, predominantly among rural workers (Shikanai-Yasuda et al. 2006). The active disease presents as two main clinical forms - the acute/subacute form (AF) and the chronic form (CF). Patients with the AF are typically children, teenagers, and young adults; they present a history of a short duration (two months in median length) and exhibit clinical manifestations characterised by the involvement of organs, such as the lymph nodes, bone marrow, liver, and spleen. CF PCM usually affects adult males who present a history of a longer duration (more than six months) and predominantly involves the lungs and upper air digestive tract (Shikanai-Yasuda et al. 2006). After treatment, numerous CF patients present sequelae, including pulmonary fibrosis (PF), emphysema (EMPH), dysphonia, and Addison's syndrome (Weber et al. 2006, Costa et al. 2013).

As observed in the case of other diseases caused by intracellular pathogens, the control of PCM depends on an effective and protective host-specific cellular immune response (Benard 2008). There are several studies on the polarisation of adaptive immune response in the case of PCM. While AF patients exhibit a Th2/Th9 profile, CF patients exhibit a Th1/Th17 profile (de Castro et al. 2013). During the treatment, the *Paracoccidioides brasiliensis-*specific cellular immunity recovers slowly, and is completely restored after successful treatment (Benard 2008).

PF and EMPH are usually observed at the time of admission of CF patients (Costa et al. 2013), and there is a wealth of emerging information which suggests that the hypoxia which results from these sequelae affects the regulatory pathways of the innate immune defence (Nizet & Johnson 2009). Few studies, pertaining to PCM, have focussed on the immune response of CF patients. For instance, it has been shown that CF patients exhibit high counts of the blood inflammatory CD14^+^CD16^++^ monocyte subset and an enhanced production of TNF-α, as estimated by monocyte cell culture (Venturini et al. 2014), after treatment. Taking into consideration recent findings that suggest that low oxygen tension in the tissues can stimulate some T cell subsets, and inhibit others (Xu et al. 2016, Doedens et al. 2013), in the present study, we aimed to evaluate the distribution of whole blood T cell subsets in PCM patients, according to the clinical forms and at different time points throughout the antifungal therapy. In addition, we quantified the serum levels of TNF-α, IL-1β, CCL3, IFN-γ, IL-4, IL-10, basic fibroblast growth factor (bFGF) and surfactant protein-D (SP-D).

## SUBJECTS AND METHODS

### Patients

Eighty-seven PCM patients from the Tropical Diseases Ward and the Systemic Mycoses Outpatient Clinic of the University Hospital, Botucatu Medical School, São Paulo State University, were studied. Patients in whom the clinical manifestations were compatible with PCM were considered as either confirmed cases or probable cases. The confirmed cases were characterised by the presence of a suggestive clinical condition and the identification of the typical *P. brasiliensis* yeast form in one or more of the clinical materials. The probable cases were characterised by the presence of suggestive clinical conditions and specific serum antibodies detected by the results of the double immunodiffusion (DID) test. The patients were classified according to their clinical forms and severity, as proposed by Mendes (1994) and the Paracoccidioidomycosis Surveillance and Control Guideline (Shikanai-Yasuda et al. 2006). Twenty-four AF patients (22 severe and two moderate cases) and 63 CF patients (18 severe, 43 moderate and two mild cases) were enrolled in this study. The exclusion criteria were as follows: presence of other systemic diseases of an infectious, inflammatory or a neoplastic source (such as co-morbidity), pregnancy and lactation. The study was approved by the local ethics committee (#283/2009-CEP). All the patients were informed of the purpose of the study, and written informed consent was obtained from all the participants.

### Moments of evaluation

All the enrolled patients were independently distributed, according to the moment of the treatment, as previously defined by Mendes (1994); this has been summarised in Supplementary data, Table. M0: before treatment. M1: under treatment, improvement of clinical manifestations, elevated erythrocyte sedimentation rates (ESRs), positive specific antibodies serum levels, as determined by the DID test. M2: under treatment, characterised by the disappearance of the initial symptomatology (clinical cure), elevated ESRs, and positive results in the DID test. M3: under treatment, clinically cured, normal ESRs and serological cure (characterised by a persistently negative serology for one year). M4: (apparent cure): clinical cure, normal ESRs, serological cure and discontinuation of the treatment (> 2 years). The distribution of the patients, according to the clinical aspects at each moment of the antifungal therapy, is shown on Table I.

**TABLE I:** Distribution of paracoccidioidomycosis patients according to the clinical aspects for each moments of antifungal therapy

Moments	Clinical forms	Levels of severity	Age in the admission	IDD - admission (1:)	IDD - sample collection (1:)	Follow-up (months)	Gender ratio (M:F)
M0 (n = 15)	Acute/Subacute (n = 7)	Severe (n = 6) Moderate (n = 1)	21(8-52)	32 (NR-512)	32 (NR-512)	–	1.3
	Chronic (n = 8)	Severe (n = 2) Moderate (n = 6)	55(35-63)	undiluted (NR-128)	undiluted (NR-128)	–	7.0
P value		0.040	0.0038	0.2091	0.2091		0.2821
M1 (n = 27)	Acute/Subacute (n = 9)	Severe(n = 8) Moderate (n = 1)	21(8-52)	32 (NR-512)	64 (NR-256)	1.0 (0.5-17)	1.2
	Chronic (n = 18)	Severe (n = 6) Moderate (n = 12)	54(35-67)	4 (NR-128)	4 (NR-64)	1.5(0.75-13)	8.0
P value		0.0128	0.0005	0.1091	0.0489	0.5618	0.1358
M2 (n = 32)	Acute/Subacute (n = 8)	Severe (n = 7) Moderate (n = 1)	24(10-85)	128 (NR-512)	2 (NR-128)	9 (2-31)	1.0
	Chronic (n = 24)	Severe (n = 5) Moderate (n = 18) Mild (n = 1)	52(35-62)	16 (NR-128)	2 (NR-16)	12 (3-47)	7.0
P value		0.0016	0.0123	0.0767	0.5776	0.3963	0.0469
M3 (n = 36)	Acute/Subacute (n = 9)	Severe (n = 9)	22(8-85)	256 (8-512)	NR	33 (14-135)	0.3
	Chronic (n = 27)	Severe (n = 8) Moderate (n = 1) Mild (n = 1)	49(29-67)	16 (NR-256)	NR	30 (5-187)	26.0
P value		< 0.0001	0.0013	0.0004	–	0.5962	< 0.0001
M4 (n = 42)	Acute/Subacute (n = 8)	Severe (n = 6) Moderate (n = 12)	26(10-38)	16 (NR-256)	NR	54 (15-102)	0.6
	Chronic (n = 34)	Severe (n = 10) Moderate (n = 23) Mild (n = 2)	46(29-72)	16 (NR-512)	NR	60 (15-180)	16.0
P value		0.0369	< 0.0001	0.8331	–	0.8854	0.0012

The comparisons were performed between acute/subacute form (AF) and chronic form (CF) patients. The comparison of levels of severity and gender ratio was performed using the Chi-Square test or the Fisher's exact test and for the others parameters, Mann-Whitney U test. Some patients were evaluated in two or more moments.

### Quantification of the peripheral blood T lymphocyte subsets

Venous blood was collected in Vacutainer tubes (BD Becton Dickinson, Franklin Lakes, NJ, USA) that contained an EDTA anticoagulant. A volume of 20 μL of TriTEST^TM^ CD3 -PerCP/CD4 -FITC/CD8-PE kit (BD, San Jose, CA, USA) and 50 μL of whole blood was added to the bead-containing TruCount^TM^ (BD). The tubes were incubated for 20 min, at room temperature, and afterwards, 450 μL of the FACS Lysing Solution was added to them. The tubes were analysed on the same day, using the BD FACSCalibur^TM^ Flow Cytometer and the MultiSet^TM^ (BD) software. The normal range of the values of TCD3^+^, TCD4^+^, and TCD8^+^, and the TCD4^+^: TCD8^+^ ratio used in this study were defined based on a study performed by Camargo (Bittencourt & Universidade Estadual Paulista 2003). In that study, 451 healthy individuals who were enrolled at the Botucatu Blood Center, whose samples were subjected to the same laboratory procedures as described above, were evaluated.

### Double agar gel immunodiffusion test

The serum levels of anti-Pb antibodies were determined through a double immunodiffusion reaction in agar gel.

### Dosage of serum mediators

The serum levels of TNF-α, IL-1β CCL3, bFGF and SP-D were quantified by the DuoSet@ ELISA Development kit (R&D systems, Minneapolis, MN, USA); and IFN-γ, IL-10 and IL-4 levels by flow cytometry, using the BD^TM^ Human Th1/Th2 Cytometric Bead Array kit (BD, Becton Dickinson, USA), according to the manufacturers' instructions. The normal range was determined based on an evaluation of 26 healthy individuals evaluated at Botucatu Blood Center.

### Statistical analysis

The comparison of two independent samples was performed using an unpaired t test or Mann-Whitney U test, and the comparison of more than two independent samples was performed using the ANOVA test with Tukey's post hoc test. The Kaplan-Meier curve was used in the analysis of the serological cure and length of treatment. The comparison of the frequencies was performed using the Chi-square test or the Fisher's exact test. The Spearman rank correlation test was used for the assessment of correlations. Statistical analyses were performed using the software SAS Version 9.3 and Graph- Pad v.5.00 software (GraphPad Software Inc, San Diego, CA, USA). The significance level was set at p < 0.05.

## RESULTS

In order to determine the counts of whole blood T cell subsets in PCM patients, AF and CF patients were categorised into five groups, according to the moments of the treatment. This categorisation was useful in both clinical and immunological evaluations, during the treatment, since each moment was based on the biological aspects of each patient, i.e., it reflected the time required for each patient to meet the established criteria.

**Fig. 1:**
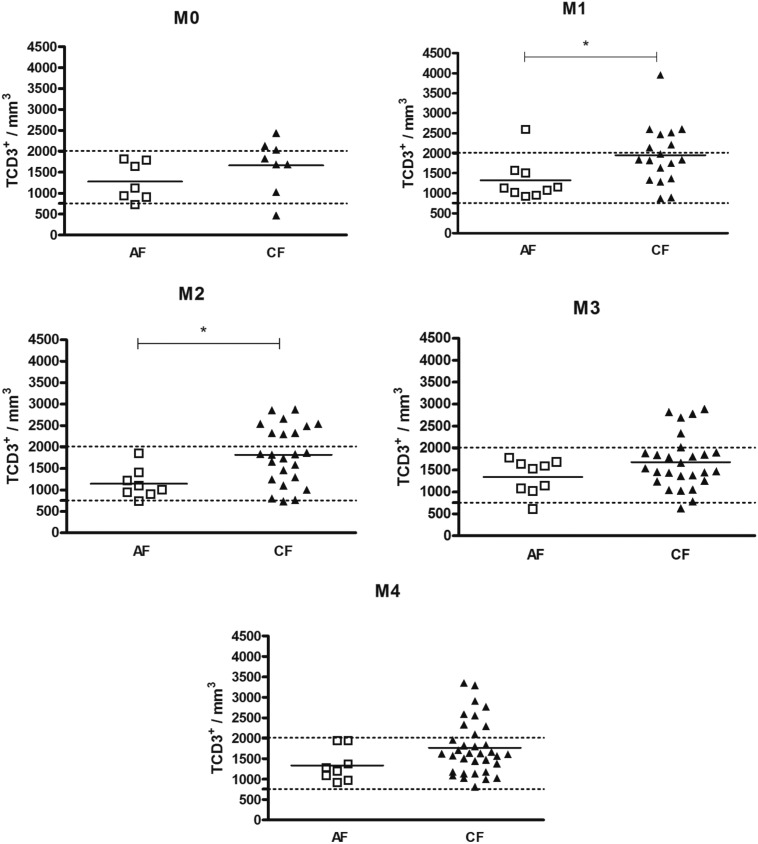
distribution of the individual counts of peripheral blood TCD3^+^ cells, of acute subacute form (AF) and chronic form (CF) patients, at different moments of the treatment. The horizontal bars indicate the average for each group. The dashed horizontal line represents the upper and lower limits of healthy individuals. Unpaired *t* test; * p < 0.05.

**Fig. 2:**
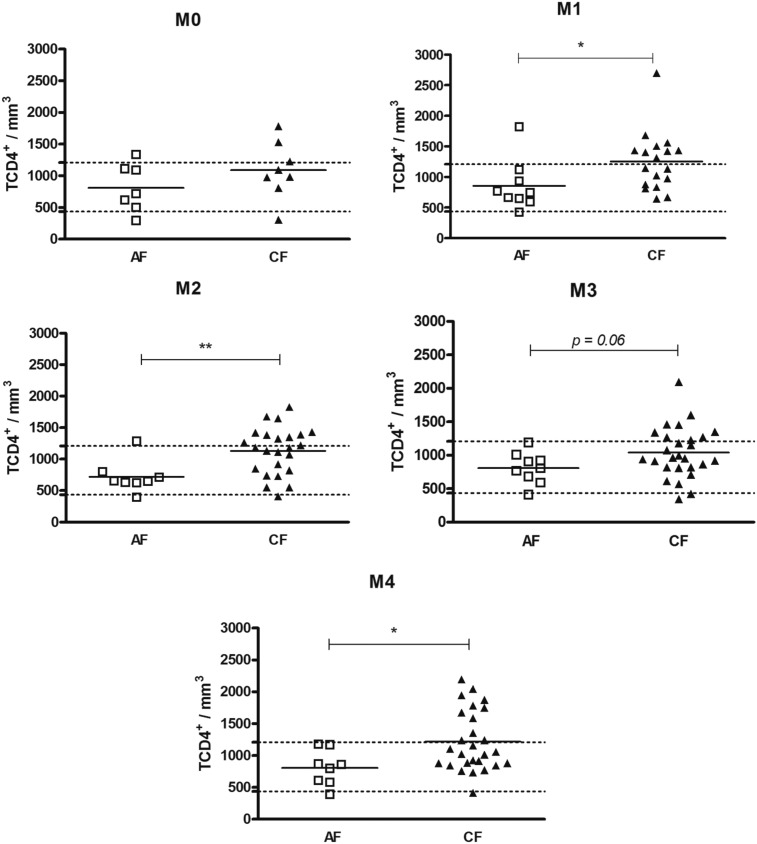
distribution of the individual counts of peripheral blood TCD4^+^ cells, of acute subacute form (AF) and chronic form (CF) patients, at different moments of the treatment. The horizontal bars indicate the average for each group. The dashed horizontal line represents the upper and lower limits of healthy individuals. Unpaired *t* test; * p < 0.05, ** p < 0.01.

**Fig. 3:**
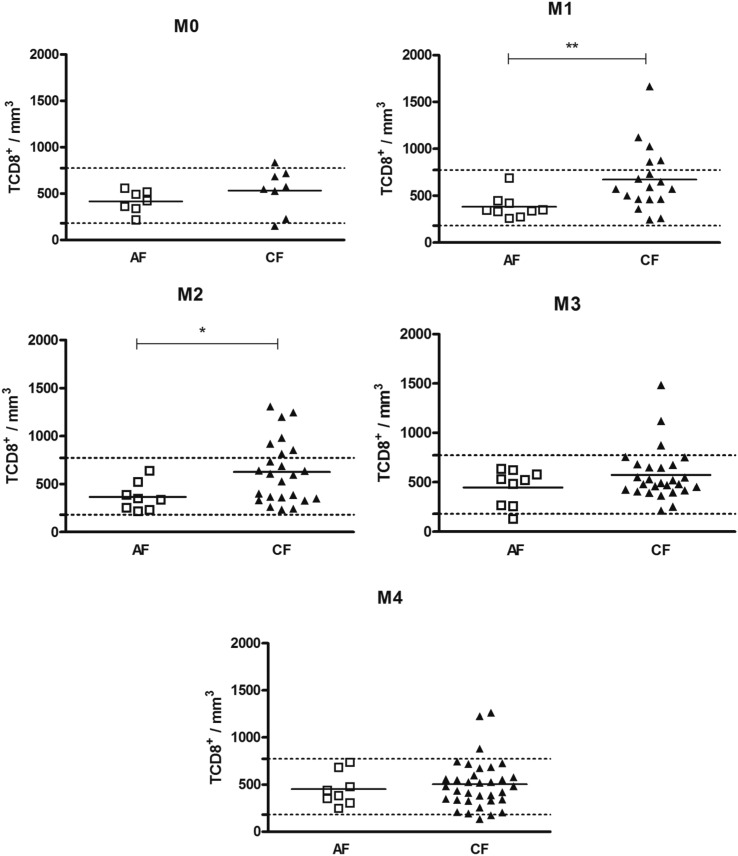
distribution of the individual counts of peripheral blood TCD8^+^ cells, of acute subacute form (AF) and chronic form (CF) patients, at different moments of the treatment. The horizontal bars indicate the average for each group. The dashed horizontal line represents the upper and lower limits of healthy individuals. Unpaired *t* test; * p < 0.05, ** p < 0.01.

First, we compared the counts between the AF and CF patients. Our findings showed that, in general, the CF patients exhibited higher counts of T cell subsets than the AF patients. Before the start of antifungal treatment (M0), there were no alterations in the counts of peripheral blood T cell subsets in both the AF and CF patients (Figs 1–3). Soon after the introduction of antifungal therapy (M1), the CF patients showed higher counts of TCD3^+^, TCD4^+^ and TCD8^+^ than the AF patients (Figs 1–3). Similarly, at M2, the CF patients showed higher counts of TCD3^+^, TCD4^+^ and TCD8^+^ than the AF patients (Figs 1–3). The CF patients, in whom an apparent cure (M4) was observed, showed a higher TCD4^+^ count than the AF patients (Fig. 2).

Next, we evaluated these counts to verify if they were in the normal range, based on data obtained from a population of healthy individuals from the same geographic region as the patients. In general, most of the AF patients showed normal counts of TCD3^+^ (Fig. 1), TCD4^+^ (Fig. 2) and TCD8^+^ (Fig. 3), as well as a normal CD4^+^/CD8^+^ ratio at all the moments of the treatment (Supplementary data, Fig. 1). A larger number of CF patients with high counts of TCD3^+^ and CD4^+^ T-cells was observed. Table II shows that the frequencies of CF patients with high counts of TCD4^+^ were higher than those of AF patients, at M2, M3 and M4.

**TABLE II:** Distribution of paracoccidioidomycosis (PCM) patients with acute and chronic forms with high counts of TCD4^+^ / total number of patients at different moments of treatment. The comparison was performed using the Chi-square test or exact Fisher test

Moments of treatment	Acute form	Chronic form	p value
M0	1/7 (14,3%)	3/8 (37,5%)	0.57
M1	1/9 (11,1%)	9/18 (50,0%)	0.09
M2	1/9 (11,1%)	11/24 (45,8%)	0.06
M3	0/9 (0,0%)	9/26 (34,6%)	0.04
M4	0/8 (0,0%)	14/32 (43,7%)	0.03

In order to clinically explain the large number of CF patients with elevated peripheral blood T cell subsets after the treatment, we analysed the distribution of the AF and CF patients with normal and elevated counts, with regards to severity, age during the blood sampling, DID serum levels at admission, and the length of the antifungal treatment. With regards to these parameters, no differences were observed between the patients with normal values and those with elevated values. Next, we analysed the time taken for the achievement of serologic cure, between CF patients with high TCD4^+^ counts, and those with normal TCD4^+^ counts. No differences were observed, based on the Kaplan-Meyer curves (Supplementary data, Fig. 2). Similarly, no differences were observed in the lengths of treatment, between CF patients with high TCD4^+^ counts and those with normal TCD4^+^ counts (Supplementary data, Fig. 3).

**Fig. 4:**
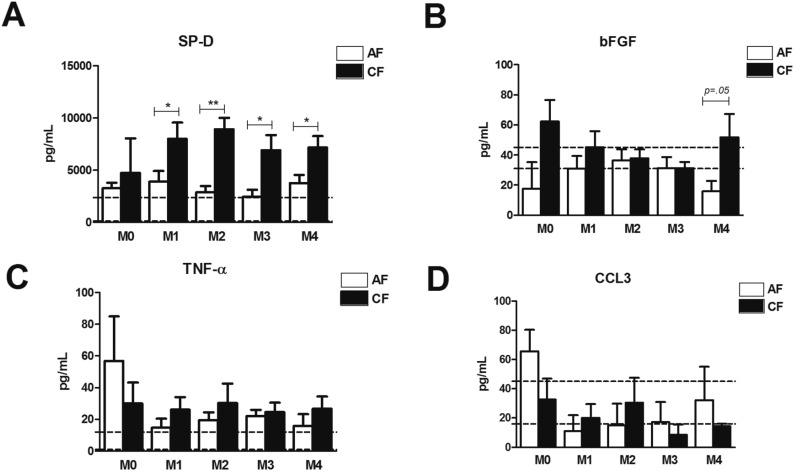
serum dosages of SP-D. bFGF. TNF-α and CCL3, of acute .subacute form (AF) and chronic form (CF) patients, at different moments of the treatment. The dashed horizontal line represents the upper and lower limits of healthy individuals. Unpaired *t* test; * p < 0.05, ** p < 0.01.

To further explore the other associations of increased TCD4^+^ counts in CF patients, we quantified the serum markers of immune response and fibrosis-related markers. Our results pointed to increased levels of SP-D in the samples of the CF patients, at M1 to M4 (Fig. 4A). No differences were observed in the levels of bFGF, TNF-α and CCL3 between the AF and CF patients (Fig. 4B, D). The levels of IL-1β, IL-10, IFN-γ and IL4 were not detectable. No correlation between the TCD4^+^ counts and all the evaluated mediators was observed (p > 0.05).

## DISCUSSION

Disparities, in terms of specific results, exist between some studies which focussed on the distribution of peripheral T lymphocyte subsets in PCM patients. The first study was carried out in non-treated patients, by Mota et al. (1988), and it showed that CF patients exhibited a decreased number of TCD4^+^ cells, while AF patients showed an increased number of TCD8^+^ cells. Bava et al. (1991) observed a decreased number of total and TCD4^+^ lymphocytes in CF patients after treatment. However, no differences were observed in the number of TCD4^+^ and TCD8^+^ cells, before (Benard et al. 1996, Cavassani et al. 2006, Bozzi et al. 2007) and after antifungal treatment (Benard et al. 1996, Bozzi et al. 2007), in CF patients. In general, these disparities may be attributed to the small number of patients enrolled (Bava et al. 1991), the type of material analysed, i.e., peripheral blood mononuclear cells (PBMCs) (Mota et al. 1988, Bava et al. 1991, Benard et al. 1996, Cavassani et al. 2006, Bozzi et al. 2007), and/or the methodology used in cell counting (Mota et al. 1988, Bava et al. 1991, Benard et al. 1996).

The results of the present study showed that there were no alterations in the peripheral whole blood TCD3^+^, TCD4^+^ and TCD8^+^ cell counts in PCM patients, before treatment, independent of the clinical forms; these findings differ from those of the first study and are in agreement with those of recent studies that evaluated T cell subsets in PBMCs. Nevertheless, we consider the lower number of patients enrolled at M0 as a limiting factor in our study.

We observed, for the first time, that CF PCM patients exhibited high peripheral blood T-lymphocyte counts (mainly in terms of TCD4^+^), soon after the introduction of antifungal therapy. The values remained high even after several years of complete and successful treatment. It is also important to highlight that, in the present study, we used an automated and standardised methodology that is widely utilised (especially in HIV/AIDS follow-up cases); this is different from previous reports. Although a few reports have addressed the adaptive immune response during the follow-up (Giannini et al. 1990, Bueno et al. 1997, Bozzi et al. 2004, 2007), this response is modified during antifungal treatment. The circulating antibody titres decrease throughout the treatment (Giannini et al. 1990, Bueno et al. 1997) and the antigen-specific cellular immunity is upregulated (Bozzi et al. 2004).

As CF PCM progresses, abnormalities in lung function and hypoxemia, predominantly involving perfusion pulmonary ventilation ratio, are observed (Afonso et al. 1979). We hypothesised that there is a possibility that these alterations occur early, since PCM patients with obstructive and mixed patterns show early indications of respiratory changes, with regards to diffusion and ventilation. Even with the regression of radiological lesions after treatment, lung function does not recover and these patients present dyspnoea during large and small efforts (Lemle et al. 1983). In response to the low levels of oxygen in the tissue, the cells attempt to restore homeostasis through the activation of a central regulatory system for hypoxia, involving hypoxia-inducible factors (HIF) (Wang et al. 1995). The activation of these transcription factors triggers several immune-related processes, including growth factor signalling, the release of proinflammatory cytokines, the expression of co-stimulatory molecules by dendritic cells, and the induced proliferation of lymphocytes (Weidemann & Johnson 2008).

Our group has observed several peripheral immunological alterations in the PCM patients, after treatment, were observed. High counts of peripheral blood CD16^++^ monocyte subsets (Venturini et al. 2014), lower counts of peripheral blood plasmocytoid dendritic cells (Venturini et al. 2017), and an enhanced expression of the NRLP3 inflammasome gene (Unpublished observations) were observed. Considering the fact that the pulmonary sequelae observed in CF PCM patients typically worsen after antifungal therapy is initiated (Costa et al. 2013), it is possible that the observed alterations occur due to hypoxemia (Tzouvelekis et al. 2007). Therefore, the expansion of peripheral blood TCD4+ cells, in the CF patients, could have been a consequence or a part of pulmonary sequelae.

Since all the CF patients in the present study exhibited PF and EMPH, and no standardised and reproducible methodology for the determination of the degree of PF is available, we quantified some serum cytokines, chemokines, growth factors and SP-D - a well-known biomarker of idiopathic pulmonary fibrosis (IPF) (Greene et al. 2002) - that could be altered in these patients and therefore, help us better understand the findings. We failed to detect the serum levels of some immunological markers, possibly due to the low sensitivity of the tests used. No alterations in the levels of CCL3, TNF-α and bFGF were observed.

However, high serum levels of SP-D were observed in the CF patients. To our best knowledge, this is the first study to quantify this member of the collect in family of C-type lectins (Pastva et al. 2007), in PCM patients. Although SP-D participates in a host's immunological surveillance mechanisms, for different microorganisms (Pastva et al. 2007), it has been used as a biomarker of IPF and is also associated with poor survival (Takahashi et al. 2006). Although a correlation with the counts of TCD4^+^ was not observed, the levels of SP-D are possibly related to hypoxemia, since increasing serum SP-D levels are seen in cases of acute lung injury (Sakamoto et al. 2012). However, the longitudinal effects of hypoxia on SP-D are not well-known.

Further studies, designed to provide clarity on the expansion of TCD4^+^ cells in CF patients, are needed, and it is essential to identify the key events involved in these mechanistic events. In addition, the use of serum SP-D as a biomarker in cases of PCM should be investigated. Although the origin and consequence of these alterations are yet to be elucidated, the results of the present study are important as they may provide new insights into the immune response of PCM patients during and after antifungal treatment. Furthermore, these findings could be used as biomarkers/predictors for the investigation of the possible alterations in the immune system of these patients.
