# Supplementary material for: Increased peripheral blood TCD4+ counts and serum SP-D levels in patients with chronic paracoccidioidomycosis, during and after antifungal therapy
Source: Mem Inst Oswaldo Cruz. 2017 Nov;112(11):748–55. doi: 10.1590/0074-02760170046 (PMC5661897; doi:10.1590/0074-02760170046)
Supplement: Supplementary file 1 [file 0074-0276-mioc-112-11-0748-Suppl01.pdf]

TABLE

Characterisation of the moments of evaluation of paracoccidioidomycosis patients as to clinical, serological and hematological parameters, and administration of antifungal drugs

Moment	Symptomatology	ESR	DID	Antifungal treatment
M_0	Present	Elevated	Positive	No
M_1	Improved	Elevated	Positive	Yes
M_2	Absent	Normal	Positive	Yes
M_3	Absent	Normal	Negative	Yes
M_4	Absent	Normal	Negative	No (> 2 years)

DID: double agar gel immunodiffusion test; ESR: erythrocyte sedimentation rate.

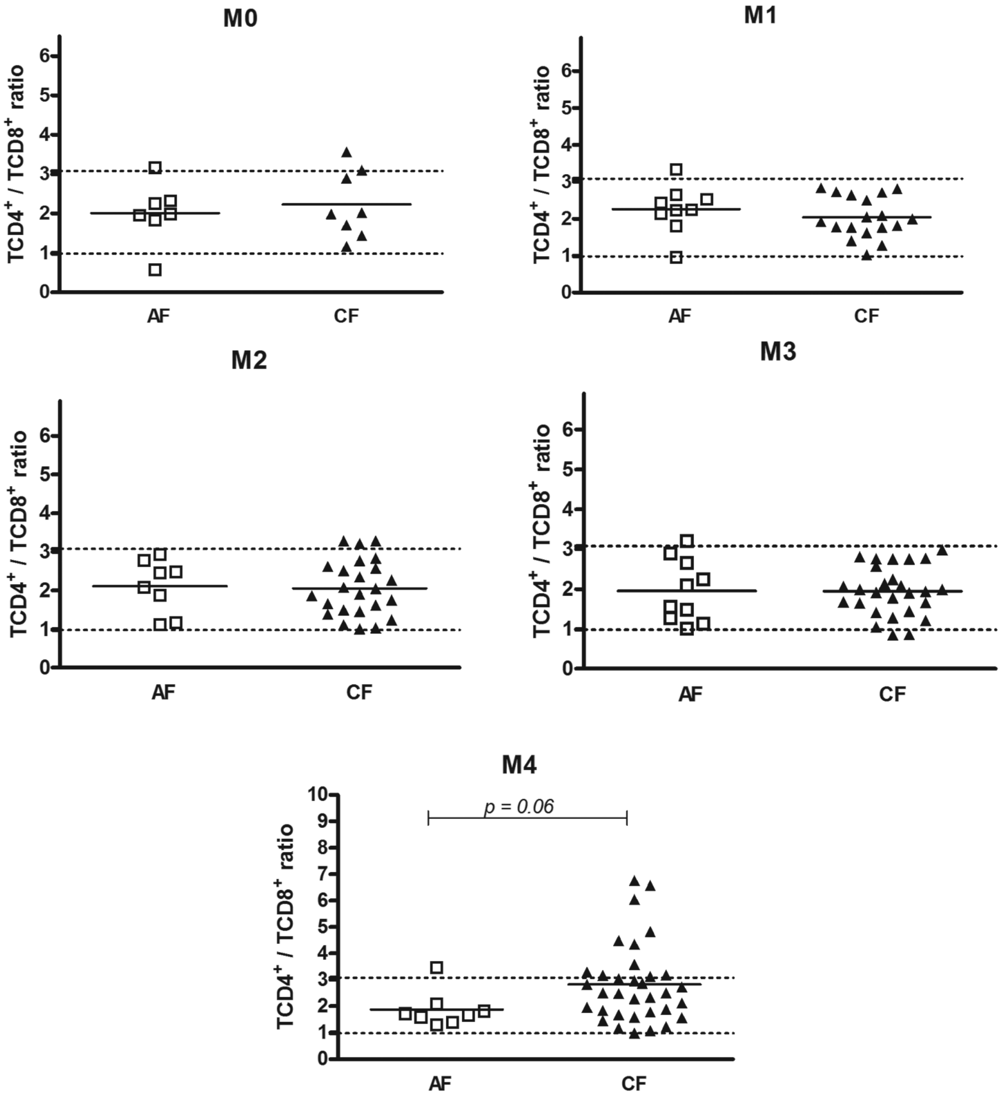

Fig. 1: distribution of the individual peripheral blood $TCD4^+/TCD8^+$ ratios, of acute/subacute form (AF) and chronic form (CF) patients, at different moments of the treatment. The horizontal bars indicate the average for each group. The dashed horizontal line represents the upper and lower limits of healthy individuals. Unpaired *t* test; * $p > 0.05$.

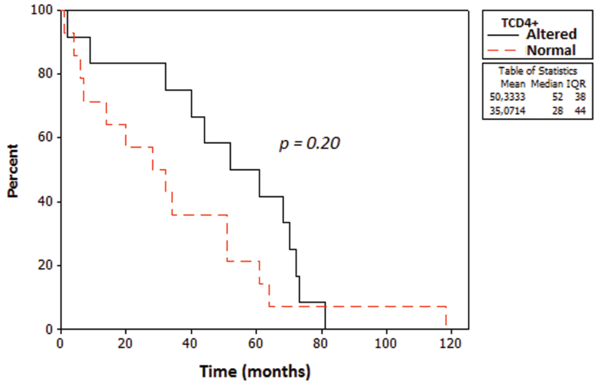

Fig. 2: Kaplan-Meier curve assessing the time taken for the achievement of serologic cure, of chronic form (CF) patients, that shows the normal (red dashed line) and higher (black line) $TCD4^+$ counts.

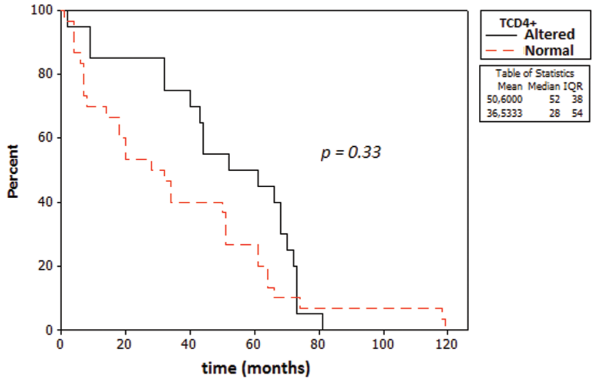

Fig. 3: Kaplan-Meier curve assessing the length of the treatment of chronic form (CF) patients, that shows the normal (red dashed line) and higher (black line) $TCD4^+$ counts.
